# Characterization of cold-active trehalose synthase from *Pseudarthrobacter* sp. for trehalose bioproduction

**DOI:** 10.1186/s40643-023-00681-0

**Published:** 2023-09-25

**Authors:** Srisakul Trakarnpaiboon, Benjarat Bunterngsook, Hataikarn Lekakarn, Daran Prongjit, Verawat Champreda

**Affiliations:** 1 Enzyme Technology Research Team, Biorefinery Technology and Bioproduct Research Group, National Center for Genetic Engineering and Biotechnology, 113 Thailand Science Park, Phahonyothin Road, Khlong Nueang, Khlong Luang, Pathumthani, 12120 Thailand; 2 Department of Biotechnology, Faculty of Science and Technology, Thammasat University, Rangsit Campus, Khlong Nueang, Khlong Luang, Pathumthani, 12120 Thailand

**Keywords:** Trehalose, Trehalose synthase, Maltose, Cold active enzyme, *Pseudarthrobacter* sp

## Abstract

Trehalose is a functional sugar that has numerous applications in food, cosmetic, and pharmaceutical products. Production of trehalose from maltose via a single-step enzymatic catalysis using trehalose synthase (TreS) is a promising method compared with the conventional two-step process due to its simplicity with lower formation of byproducts. In this study, a cold-active trehalose synthase (PaTreS) from *Pseudarthrobacter* sp. TBRC 2005 was heterologously expressed and characterized. PaTreS showed the maximum activity at 20 °C and maintained 87% and 59% of its activity at 10 °C and 4 °C, respectively. The enzyme had remarkable stability over a board pH range of 7.0–9.0 with the highest activity at pH 7.0. The activity was enhanced by divalent metal ions (Mg^2+^, Mn^2+^ and Ca^2+^). Conversion of high-concentration maltose syrup (100–300 g/L) using PaTreS yielded 71.7–225.5 g/L trehalose, with 4.5–16.4 g/L glucose as a byproduct within 16 h. The work demonstrated the potential of PaTreS as a promising biocatalyst for the development of low-temperature trehalose production, with the advantages of reduced risk of microbial contamination with low generation of byproduct.

**Graphical abstract:**

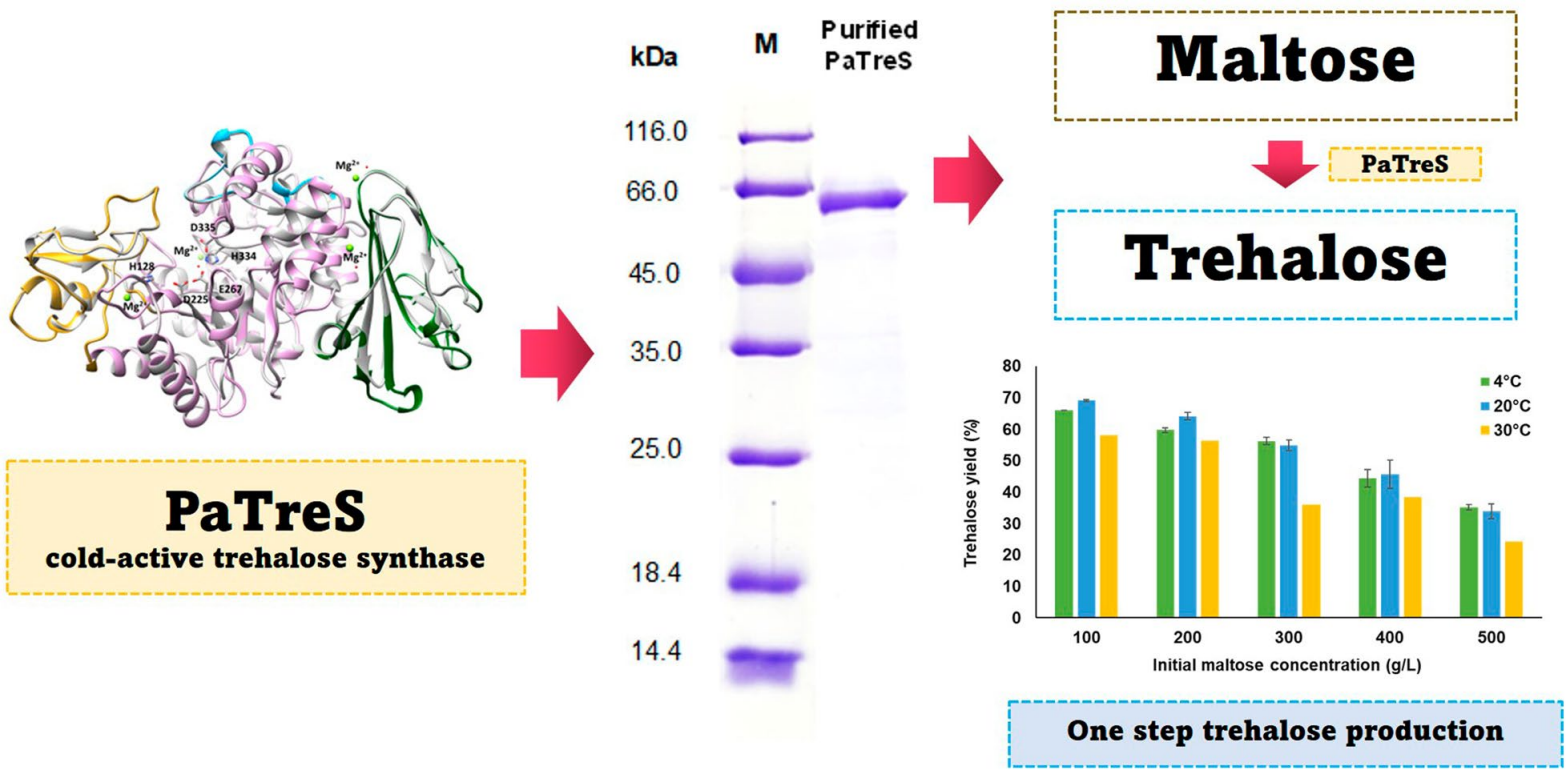

**Supplementary Information:**

The online version contains supplementary material available at 10.1186/s40643-023-00681-0.

## Introduction

Trehalose, a naturally occurring colorless and odorless sugar, serves as a potent additive to protect functional biological compounds in diverse consumable products from adverse environmental conditions. It is accepted as a safe food ingredient by the US Food and Drug Administration (Ohtake and Wang 2011). Nowadays, trehalose is widely used as a functional ingredient not only in the food application but also in the cosmeceutical and pharmaceutical industries as a functional additive, stabilizer, humectant, moisturizing agent, and sweetener purposes (Burek et al. 2015; Chang et al. 2010).

Trehalose is basically a disaccharide comprising two α-glucose molecules connected with α-1,1-glycosidic linkage (Schiraldi et al. 2002). Currently, trehalose is produced via a two-step enzymatic method from starch employing the enzymes maltooligosaccharide trehalose synthase (TreY) and maltooligosyltrehalose trehalohydrolase (TreZ). However, this approach has certain drawbacks because it produces a variety of byproducts, such as branched starch, maltose, and maltotriose due to the specificity of TreY that performs the catalysis against only linear maltooligosaccharides with chain length ≥ 3 (Cai et al. 2018).

A single-step trehalose biosynthesis by trehalose synthase (TreS) has been proposed as a promising alternative (Cai et al. 2018). TreS (EC 5.4.99.16) is an isomerase that catalyzes the reversible single-step enzymatic reaction in transformation of maltose (α-D-glucopyranosyl-(1 → 4)-α-D-glucopyranoside) to trehalose (α-D-glucopyranosyl-(1 → 1)-α-D-glucopyranoside) through transglucosylation and hydrolytic activities (Cai et al. 2019). According to the CAZy database, TreS is categorized into glycoside hydrolase family (GH) 13 subfamily 16 and 33 (http://www.cazy.org/) (Janecek and Gabrisko 2016; Stam et al. 2006). The enzymes have been identified in several bacterial genera, e.g., *Pseudomonas* sp. (Gao et al. 2013; Lee et al. 2005; Trakarnpaiboon et al. 2021), *Thermus* sp. (Koh et al. 1998; Lin et al. 2020; Zdziebło and Synowiecki 2006), *Deinococcus* sp. (Filipkowski et al. 2012; Wang et al. 2007), *Arthrobacter sp.* (Cai et al. 2019) and *Enterobacter* sp. (Yue et al. 2009). Most reported TreS enzymes showed an optimal temperature at 25–50 °C with approximately 60–74% of conversion yield under specified experimental conditions. At higher temperatures, the flexibility of the protein increases making the catalytic pocket more straightforward to hydrolytic reaction, and hence, decreases the yield of trehalose while increasing thess glucose side product (Cai et al. 2018; Koh et al. 2003). On the contrary, trehalose production by TreS at low temperatures may lead to decreased byproduct (glucose) generation. Despite cold-active enzymes or psychrophilic enzymes, which exhibited higsh specific activity at temperature range below 25 °C, have been discovered and utilized in various applications (Hamid et al. 2022; Joseph et al. 2008; Kuddus 2018; Maharana and Ray 2015; Santiago et al. 2016; Sarmiento et al. 2015), cold-active TreS is still uncommon (Cai et al. 2018). In this work, a cold-active recombinant TreS from *Pseudarthrobacter* sp. TBRC 2005 (PaTreS) was characterized. Application of PaTreS on trehalose production under low-temperature conditionss was demonstrated, showing its potential for further implementation in industry.

## Materials and methods

### Bacterial strains, plasmids, cultures, and chemicals

*Pseudarthrobacter* sp. TBRC 2005 was provided by the Thailand Bioresource Research Center (TBRC) (www.tbrcnetwork.org). The bacterial strain was cultivated at 30 °C in nutrient broth for genomic DNA extraction. *Escherichia coli* DH5α was used as a host for plasmid propagation and *E. coli* BL21(DE3) (Invitrogen, Carlsbad, CA, USA) was used for recombinant protein production. The pJET1.2 vector and pET28a vectors (Novagen, Darmstadt, Germany) were used for cloning and protein expression, respectively. Maltose and trehalose used as standardss for analysis were purchased from Megazyme (Wicklow, Ireland). All analytical-grade chemicals and reagents used in this work were purchased from Sigma-Aldrich, Merck and Fluka.

### DNA extraction, PaTreS gene isolation, and plasmid construction

To isolate the *PaTreS* gene, the genomic DNA of *Pseudarthrobacter* sp. TBRC 2005 was extracted using GeneJET genomic DNA purification kit (Thermo Scientisfic, Inc., Waltham, MA, USA). The *PaTreS* gene was amplified from the genomic DNA using the universal primers designed based on Cai et al. (2019). The target PCR product was subsequently purified and cloned into pJET1.2/blunt vector (Thermo Scientific, Waltham, MA, USA). After propagation in *E. coli* DH5α, the recombinant plasmid was extracted and subjected tso DNA sequencing to determine nucleotide sequence of the *PaTreS* gene (1st BASE Laboratories Sdn. Bhd., Malaysia).

For recombinant plasmid construction, the *PaTreS* gene was amplified from pJET1.2/*PaTreS* using specific primers, ArTreS/EcoRI (5′-GCGAATTCATGATTTTCAATCCGCAAGACTCG-3′) and ArTreS/NotI (5′-GCGGCCGCATTCTCAATCGACAGGATCGGCAT-3′) containing sites for restriction enzymes (underlined). Following digestion, the gene fragment was ligated to the pET28a vector at the EcoRI and NotI cloning sites to produce PaTreS-pET28a, which was subsequently introduced into *E. coli* DH5 using the heat-shock method (Sambrook and Russell 2001). The transformants were selected on Luria–Bertani (LB) agar (tryptone 10 g/L, yeast extract 5 g/L, and NaCl 5 g/L) supplemented with 50 µg/mL kanamycin and incubated at 37 °C for 18 h. The transformants were verified by colony PCR using PaTreS/EcoRI and PaTreS/NotI-specific primers. After confirmation of nucleotide sequence, the *PaTreS*-pET28a wass then transformed into *E. coli* BL21(DE) used as an expression host. The transformants were selected on LB agar containing 50 μg/mL kanamycin at 37 °C. The selected transformants were subsequently verified by colony PCR.

### Sequence and structural analysis

The sequence similarity analysis of PaTreS was analyzed against the NCBI database (https://blast.ncbi.nlm.nih.gov/Blast.cgi; accessed on 4 March 2022). The signal peptide for protein translocation was predicted using SignalP 5.0 (https://services.healthtech.dtu.dk/ service.php?SignalP-5.0; accessed on 4 March 2022). The three-dimensional structure of the enzyme was predicted using SWISS-MODEL homology modeling (Schwede et al. 2003) based on the trehalose synthase from *Thermobaculum terrenum* (PDB 5 × 7u) (Wang et al. 2017). The predicted model was visualized using Chimera software (Pettersen et al. 2004).

### Production and purification of recombinant PaTreS

To produce recombinant PaTreS, *E. coli* BL21(DE3) harboring *PaTreS*-pET28a was cultivated in LB broth supplemented with 50 μg/mL kanamycin at 37 °C with shaking at 200 rpm for 18 h for inoculum preparation. The inoculum was then transferred at 1% v/v into LB broth containing 50 μg/mL kanamycin and cultivated at 37 °C, 200 rpm for 3 h. After that, β-D-1-thiogalactopyranoside (IPTG) (0.5, 0.25, 0.1 mM) was added to induce expression of the PaTreS gene and the cells were further cultivated at 25 °C for 3 h. The cells were collected by centrifugation at 8000 × *g* for 10 min at 4 °C. The cell pellet was resuspended in 50 mM sodium phosphate buffer pH 7.4 and disrupted by sonication using Ultrasonic Processor model VCX 130 PB (Sonics & Materials, Inc., Newtown, CT, USA). After that, cell debris and insoluble protein were removed by centrifugation at 12,000 × *g* for 30 min.

The His trap™ FF affinity chromatographic column was used to purify the target protein according to the manufacturer's instructions (GE Healthcare, St Giles, UK). The unbound proteins were washed using a washing buffer (20 mM sodium phosphate buffer pH 7.4, 0.5 M NaCl, containing 20 mM and 50 mM imidazole, respectively). Subsequently, the elution step was carried out by step-gradient elution with the buffer containing 100, 200, 300, and 400 mM imidazole, respectively. The purified protein was pooled and desalted using an Amicon® centrifugal filter with a 30 kDa molecular weight cut-off (Merck Millipore Ltd., Cork, Ireland). The purified protein was then analyzed by 12% SDS–polyacrylamide gel electrophoresis. Protein concentrations were determined by Bio-Rad protein assay kit (BioRad Laboratory, Hercules, CA, USA) using bovine serum albumin as a standard.

### Biochemical characterization of PaTreS

To understand the biochemical properties of recombinant PaTreS, the effects of pH, temperatures, metal ions, pH stability, and thermostability were investigated. The effect of pH on trehalose synthase activity of purified PaTreS was determined at 40 °C in 50 mM sodium acetate buffer (pH 3.0–5.0), potassium phosphate buffer (pH 6.0–8.0), and glycine–NaOH buffer (pH 9.0–11.0). Before measuring activity, the enzyme was pre-incubated at 30 °C for 1 h at several pH ranging from 3.0 to 11.0 to test the pH stability of PaTreS. The srelative activity of an enzyme was defined as 100% in the absence of pre-incubation.

The effect of temperature was evaluated in 50 mM phosphate buffer pH 7.0 under temperatures ranging from 4 to 70 °C. The maximum activity obtained was set as 100% relative activity. The enzyme was pre-incubatsed at 4–70 °C for 6 h in 50 mM phosphate buffer (pH 7.0) before its activity was measured to test its thermostability. The activity of the enzyme without pre-incubation was defined as 100% relative activity.

The effects of metal ions on enzyme activity were investigated using demetallized enzyme in the presence of 0.1, 1, 10, and 20 mM final concentrations of CuCl_2_, MgCl_2_, MnCl_2_, CoCl_2_, FeCl_2_, ZnCl_2_, LiCl, NiCl_2,_ and CaCl_2_. The demetallized enzyme was prepared by treating the purified PaTreS with 30 mM EDTA for 30 min at 30 °C before removing EDTA by an sextensive buffer exchange membrane centrifugation tube (MWCO 10 kDa, Pall Corporation, Port Washington, USA). The relative activity was determined by comparing the activity in the presence of metal ions to the obtained in the absence of metal ions.

The TreS activity was determined using maltose as the substrate according to Trakarnpaiboon et al. 2021 with slight modification. Reactions contained 50 µl of purified PaTreS and 250 μl of 5% (*w*/*v*) maltose solution in 50 mM phosphate buffer pH 7.0. The reaction was incubated at 40 °C for 1 h before measurement of the generated product by Trehalose Assay Kit (Megazyme). One TreS unit is defined as the amount of enzyme to produce 1 µmol/min of trehalose from maltose.

### Substrate specificity and kinetic parameters

The specificity and kinetic parameters of PaTreS were examined against various substrates including maltose, trehalose, sucrose, glucose, fructose, and lactose. To evaluate the substrate specificity, the purified PaTreS was mixed with 2% (*w*/*v*) substrate dissolved in 50 mM phosphate buffer pH 7.0. The reaction was incubated at 30 °C for 3 h. The sugar composition was determined using high-performance liquid chromatography.

Kinetic parameters were determined using varying concentrations of maltose and trehalose (0–1500 mM) as the substrates. The enzyme reaction rate was measured at 20 °C and pH 7.0. The Lineweaver–Burk equation was used to obtain the kinetic values (Km and Vmax) for each substrate. All experiments were performed in triplicate.

### Effect of enzyme loading and substrate concentrations

The influence of PaTreS loading on trehalose yield and specificity was investigated. The reaction mixture contained 100 g/L maltose in 50 mM phosphate buffer pH 7.0 with a varying amount of purified PaTreS (1–4 mg/g substrate). The effect of substrate concentrations on enzyme activity was tested using 100, 200, and 300 g/L of maltose with 2 mg protein of PaTreS per g substrate. The reactions were incubated at 20 °C for 15 h. The products were analyzed using high-performance liquid chromatography.

### Bioproduction of trehalose from maltose

The purified PaTreS (2 mg protein/g substrate) was added into 50 mL of 100–300 g/L maltose dissolved in 50 mM phosphate buffer pH 7.0. The reaction mixtures were incubated at 20 °C with a mixing rate of 50 rpm. The samples were collected at regular intervals during the 20 h incubation. The enzymatic reaction was stopped by heating at 100 °C for 10 min. The amount of trehalose was analyzed by high-performance liquid chromatography.

### Product analysis

The concentrations (%, *w*/*v*) of products (glucose, maltose, and trehalose) released from PaTreS-catalyzed reactions were determined at 40 °C using a high-performance liquid chromatograph model LC-20A Series (Shimadzu-GL Sciences, Kyoto, Japan) using Shodex HILICpak VG-50 4E column (4.6 250 mm, 5 m). Acetonitrile:ultrapure water (80:20 *v*/*v*) was used as the mobile phase at a flow rate of 1.0 mL/min.

## Results

### Identification of PaTreS and structural analysis

The gene encoding trehalose synthase (PaTreS) was successfully isolated from the genomic DNA of *Pseudarthrobacter* sp. TBRC 2005 using TreS universal primers designed based on Cai et al. (2019). The size of the *PaTreS* gene was 1794 bp which encoded 597 amino acid residues without a signal peptide. The calculated molecular weight and pI value of the protein are 67.94 kDa and 4.75, respectively. The gene exhibited the highest similarity to maltose alpha-D-glucosyltransferases from unclassified *Pseudarthrobacter* sp. (WP_141158082.1) (99% identity), followed by *Arthrobacter* sp. ISL-85 (WP_214945216.1) (98% identity), and *Arthrobacter* sp. NicSoilC5 (98% identity).

**Fig. 1:**
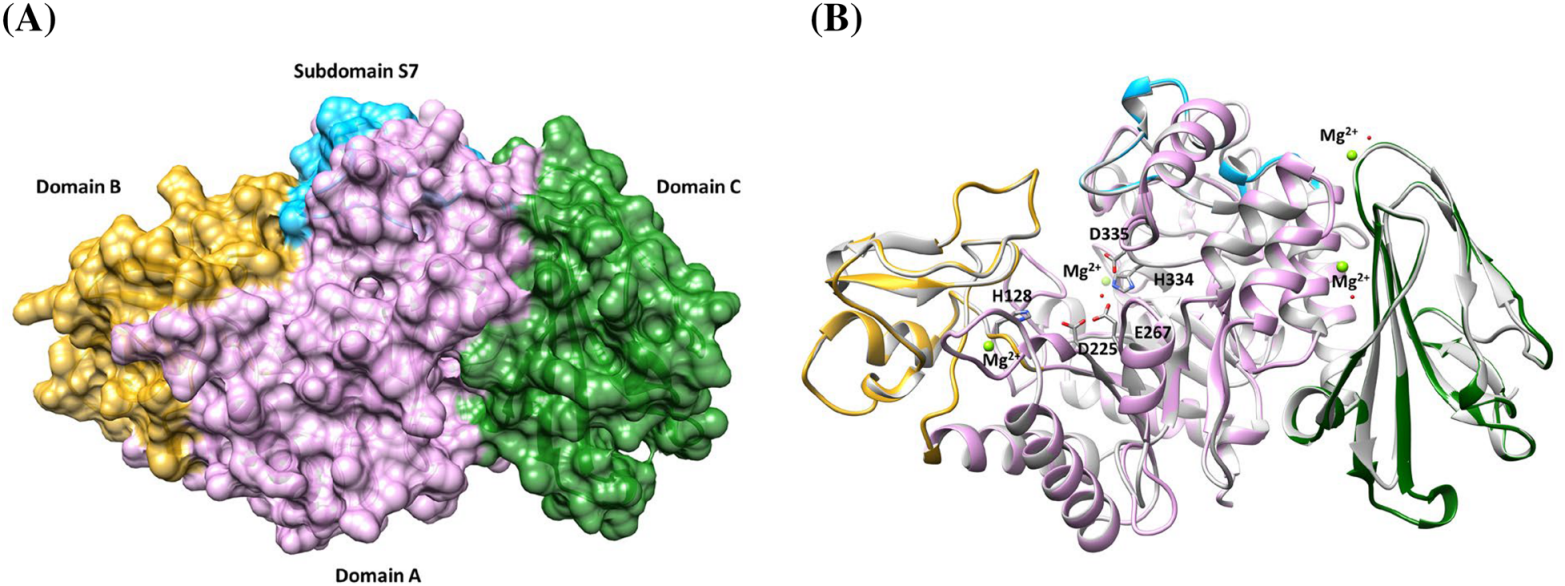
Overall predicted three-dimensionals structure of PaTreS. **A** Cartoon representation of four distinct domains of ArTreS including domain A, B, C, and subdomain S7 represented in pink, yellow, green, and blue, respectively. **B** The superimposition of PaTreS and TtTS from *Thermobaculum terrenum* (PDB 5 × 7u) represents the proposed catalytic residues (H128/D225/E267/H334/D335) and Mg^2+^ ions

Using SWISS-MODEL homology modeling, the three-dimensional structure of PaTreS was predicted based on the crystal structure of TreS from *Thermobaculum terrenum* (PDB 5 × 7u), which shared 57.95% identity and a GMQE score of 0.79. The predicted 3D-structure of PaTreS contained 4 domains comprising domain A, domain B, Domain C, and the subdomain S7 (Fig. 1A). Domain A (27–125, 202–353 and 379–477) located at the N-terminus is a catalytic module containing the TIM barrel structure. The proposed key amino acid residues are consisted of H128/D225/E267/H334/D335 (Agarwal and Singh 2021) (Fig. 1B). Domain B (126–201) is inserted between the TIM barrel structure. Domain A and B are connected via the subdomain S7 (354–378). Domain C (478–582) containing all the β-strands is located at the C-terminus. PaTreS was assigned to the GH13 16 subfamily based on phylogenetic analysis (Additional file 1: Fig. S1). Furthermore, sequence logo analysis revealed that PaTreS contains five conserved regions that are similar to those found in GH13 16 subfamily TreS enzymes (Additional file 1: Fig. S2).

### Expression and characterization of PaTreS

**Fig. 2:**
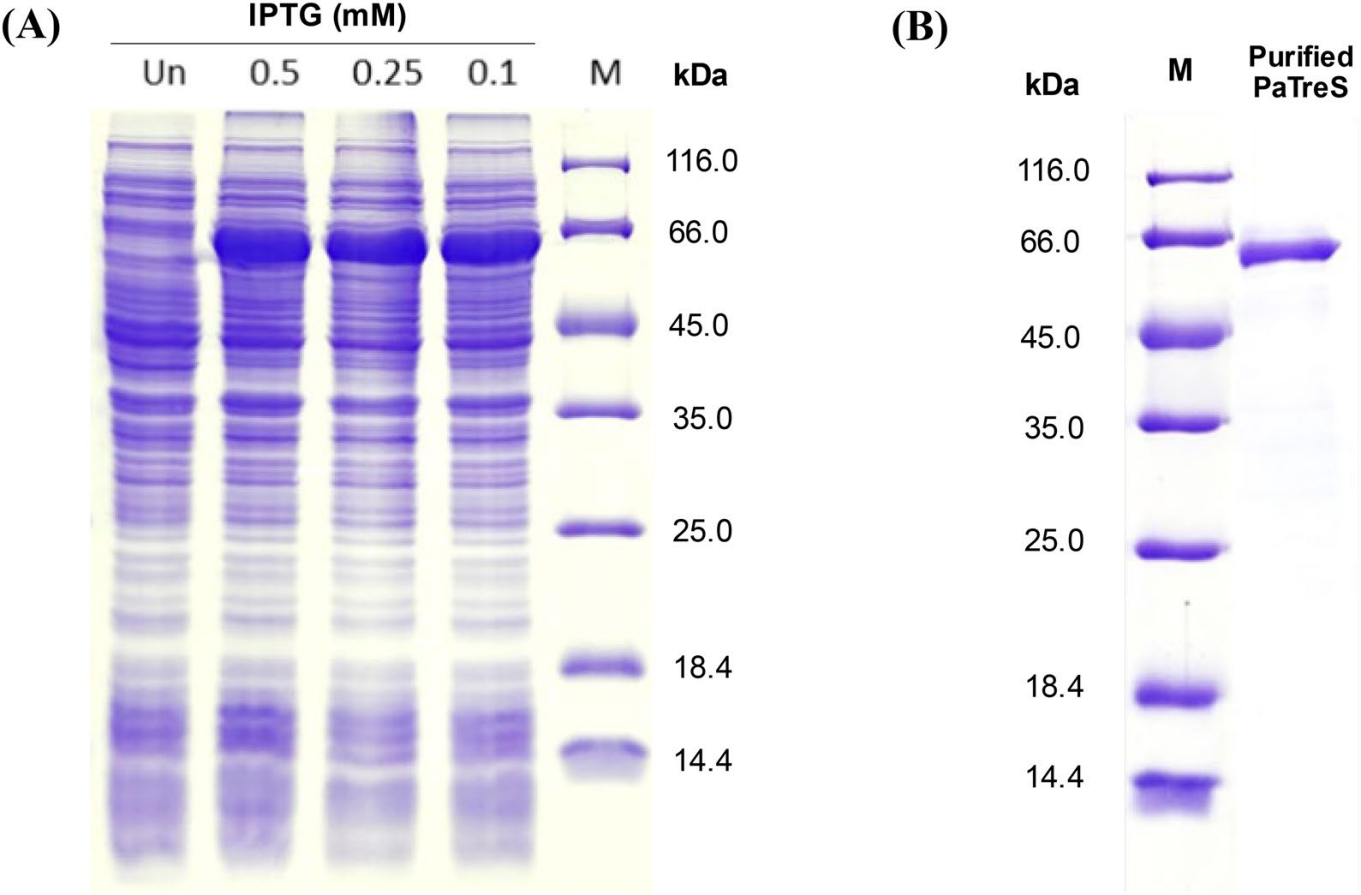
SDS-PAGE analysis of recombinant PaTreS expression in *E. coli* BL21(DE3) **A** and PaTreS purification **B**. The recombinant strain was cultivated at 25 °C and induced with 0.1, 0.25, and 0.5 mM IPTG for 3 h. Lane M: standard protein marker

After induction by 0.1–0.5 mM IPTG at 25 °C for 3 h, the *PaTreS* gene was expressed in *E. coli* BL21 (DE3) and resulted in production of the recombinant protein in an active soluble form (Fig. 2A). The SDS-PAGE analysis showed that the recombinant PaTreS was purified to more than 90% homogeneity using Ni^2+^-affinity column, corresponding to an apparent molecular weight of 66 kDa (Fig. 2B).

**Fig. 3:**
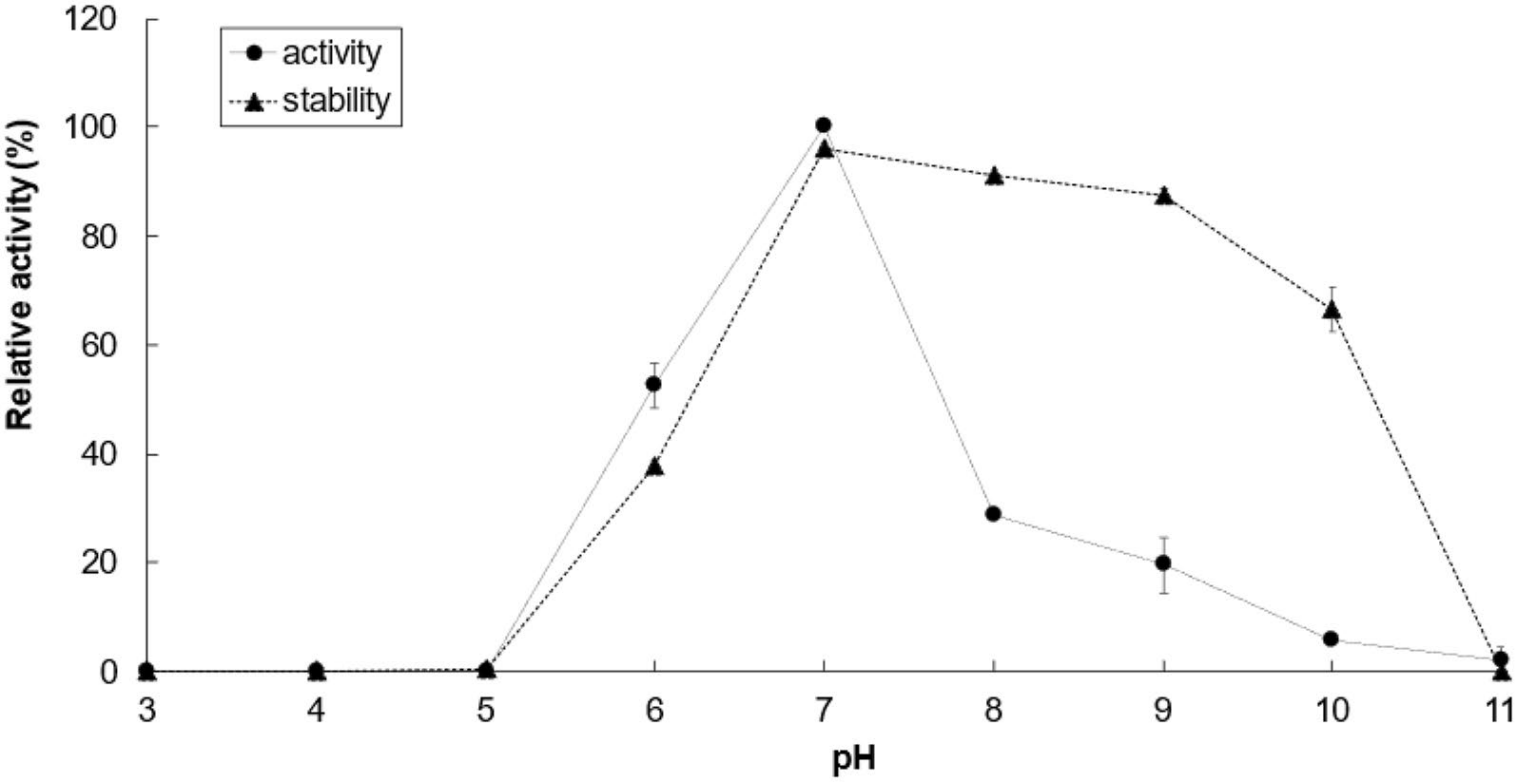
Effects of pH on the activity and stability of PaTreS with maltose as substrate. The 50 mM sodium acetate (pH 3.0–5.0), 50 mM potassium phosphate (pH 6.0–8.0), and 50 mM glycine–NaOH (pH 9.0–11.0) were used to assay the enzyme activities at various pH values (pH 4.0–11.0). To examine pH stability, the enzymes were incubated at various pH values (pH 4.0–11.0) at 30 °C for 1 h. The residual activities were measured at pH 7.0 using 1% (w/v) maltose as a substrate

**Fig. 4:**
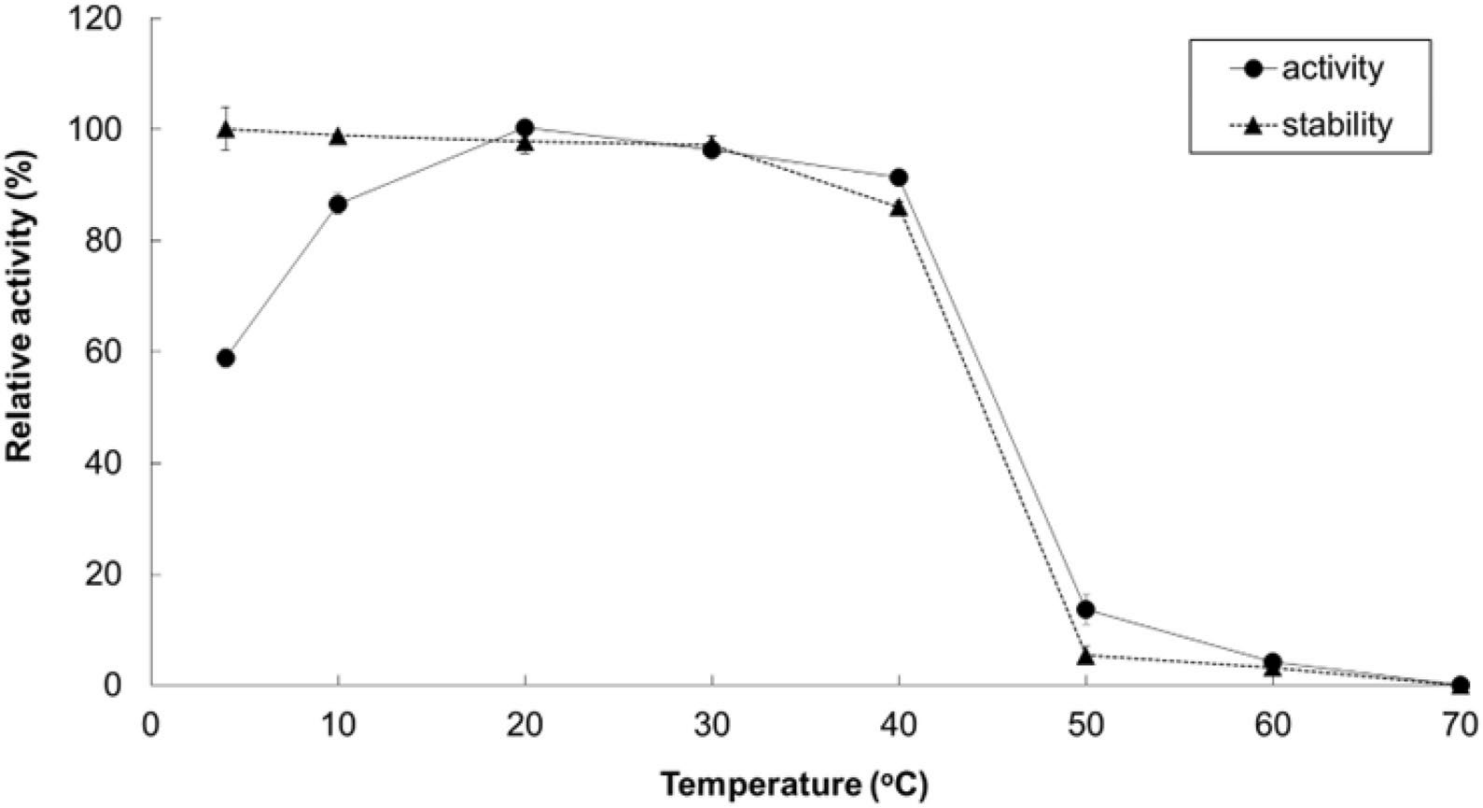
Effects of temperature on the activity and stability of PaTreS with maltose as substrate. Enzyme activities at various temperatures (4–70 °C) were assayed. To examine the thermal stability, the enzymes were incubated at various temperatures (4–70 °C) for 6 h and then were immediately cooled. The residual activities were measured at 40 °C

Recombinant PaTreS exhibited the maximum activity at pH 7.0, but its catalytic activity markedly decreased at pH below 6.0 and above 8.0 with 52.6% and 28.6% of residual activity, respectively (Fig. 3). However, the enzyme had high stability in the pH range of 7.0–9.0 after 6 h of pre-incubation (Fig. 3). According to analysis of activity at 4–70 °C, PaTreS showed the highest activity at 20 °C (Fig. 4). Interestingly, 58.9% and 86.6% of the maximum activity was observed at the temperature of 4 and 10 °C, respectively. The enzyme showed high thermostability with > 90% remaining activity after pre-incubation at 4–40 °C (Fig. 4). The enzymatic activity markedly decreased at a temperature above 40 °C.

**Fig. 5:**
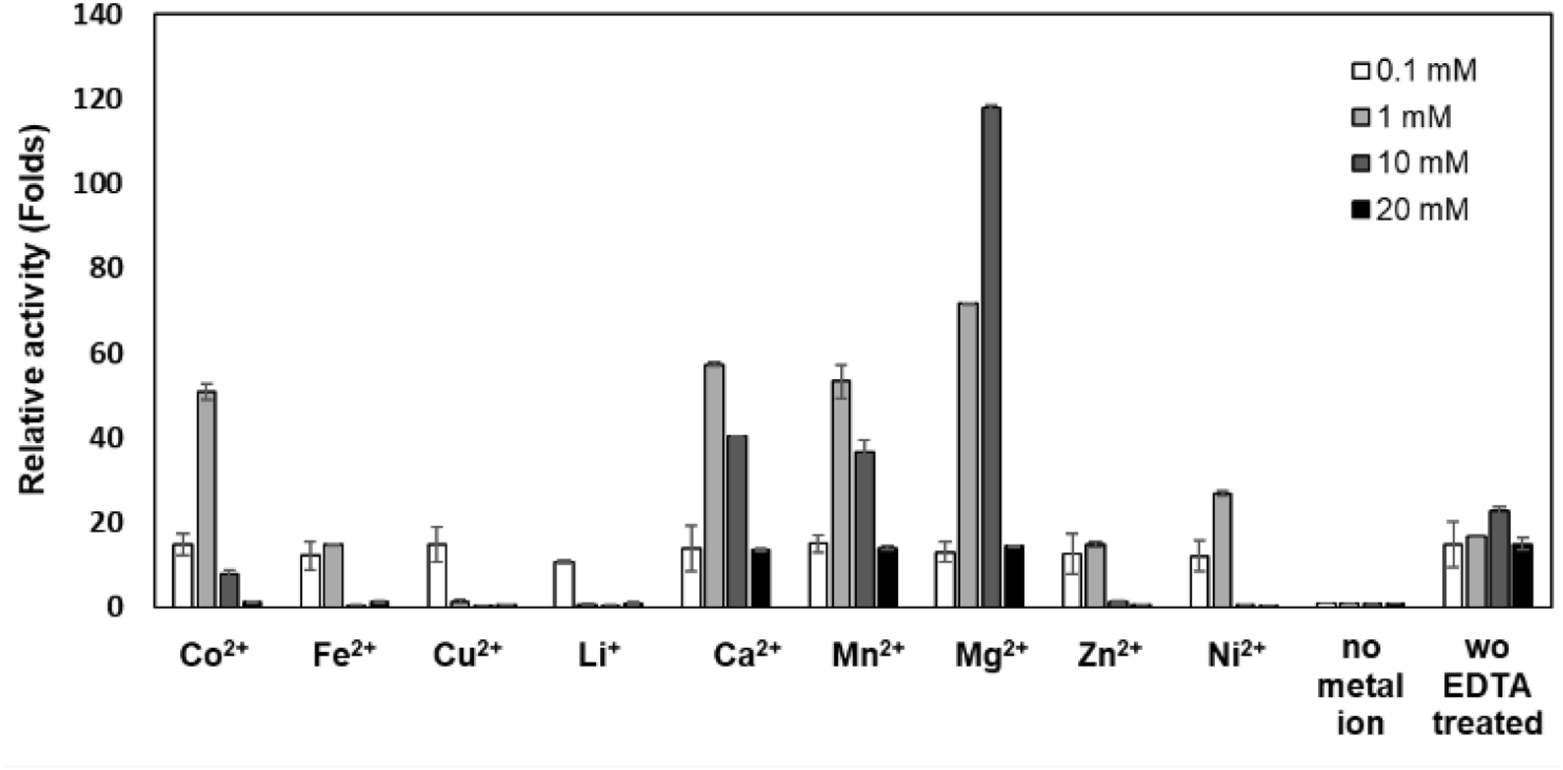
Effects of metal ions on PaTreS activity. The enzyme was incubated with 0.1, 1, 10, and 20 mM of different metal ions at 40 °C in 50 mM phosphate buffer pH 7

Additionally, the impact of metal ions on PaTreS activity was investigated (Fig. 5). The activity of the enzyme was decreased by the chelation of metal ions from the enzyme. Diverse divalent metal ions restored and enhanced the activity to varying degrees. PaTreS activity was strongly increased by Mg^2+^, Ca^2+^, and Mn^2+^ at concentrations of 1 mM and 10 mM. The highest enzyme activity was achieved by the addition of Mg^2+^ at 10 mM. However, at a concentration of 0.1 mM and 20 mM, almost all examined metal ions decreased PaTreS activity.

### Substrate specificity and kinetic parameters

The specificity of PaTreS was investigated on a variety of sugars, including maltose, trehalose, lactose, sucrose, fructose, and glucose under the optimal enzyme working conditions. According to the results, PaTreS selectively facilitated the reversible conversion of maltose and trehalose by releasing a small amount of glucose as the byproduct (Table 1). There was no evidence of catalytic activity against other sugars. Regarding kinetic performance, PaTreS exhibited the highest rate of reaction against maltose with a *V*_*max*_ of 6.74 ± 0.26 mM/min, followed by trehalose (*V*_*max*_ 5.70 ± 0.38 mM/min) with the *K*_*m*_ 285.01.76 ± 3.30 and 808.14 ± 1.16 mM for maltose and trehalose, respectively. This corresponded to a turnover number (*k*_*cat*_) of 2.24 s^−1^ and catalytic efficiency (*k*_*cat*_/*K*_*m*_) of 126.96 on the conversion of maltose to trehalose.

**Table 1:** Substrate specificity and kinetic analysis of PaTreS

Substrate	Product	Byproduct	K_m_ (mmol/l)	V_max_ (U/mg)
Maltose	Trehalose	Glucose	369.76 ± 2.45	6.622 ± 0.34
Trehalose	Maltose	Glucose	840.95 ± 1.38	1.66 ± 0.68

^*^ND = not detected

### Effect of enzyme loading and substrate concentration on reaction specificity

**Fig. 6:**
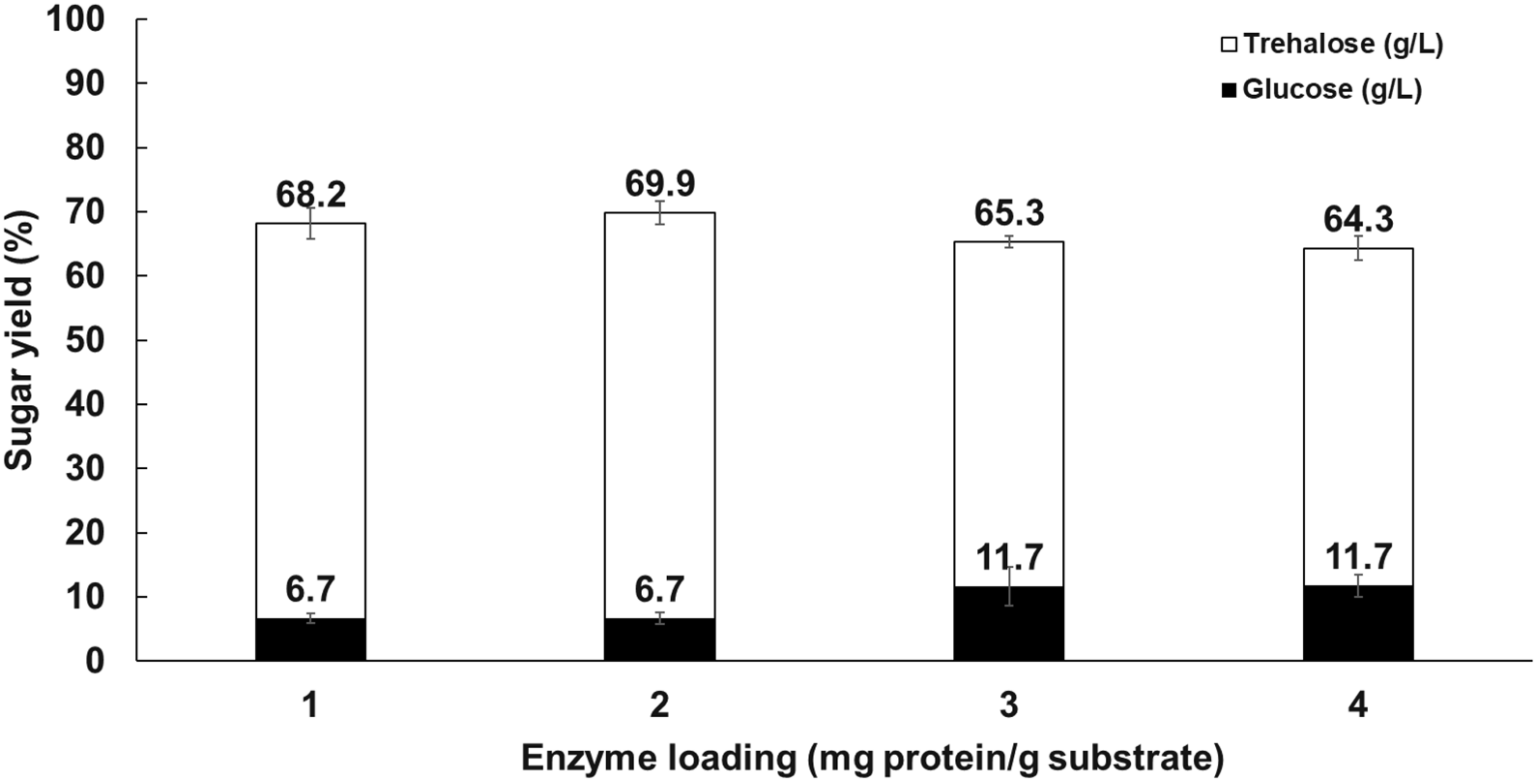
Effects of enzyme loading on trehalose production by PaTreS. The reactions were carried out in 50 mM phosphate buffer (pH 7.0) at 20 °C for 24 h

The effect of enzyme loading on trehalose production yield and by-product formation was investigated by varying PaTreS enzyme loading ranging from 1 to 4 mg protein/g substrate using 100 g/L maltose as a substrate (Fig. 6). Based on the results, the greatest trehalose yield of 69.9% with 6.7% of glucose as a by-product was obtained with an enzyme dosage of 2 mg/g. Increasing enzyme dosage decreased trehalose production yields while boosting glucose by-product formation.

**Fig. 7:**
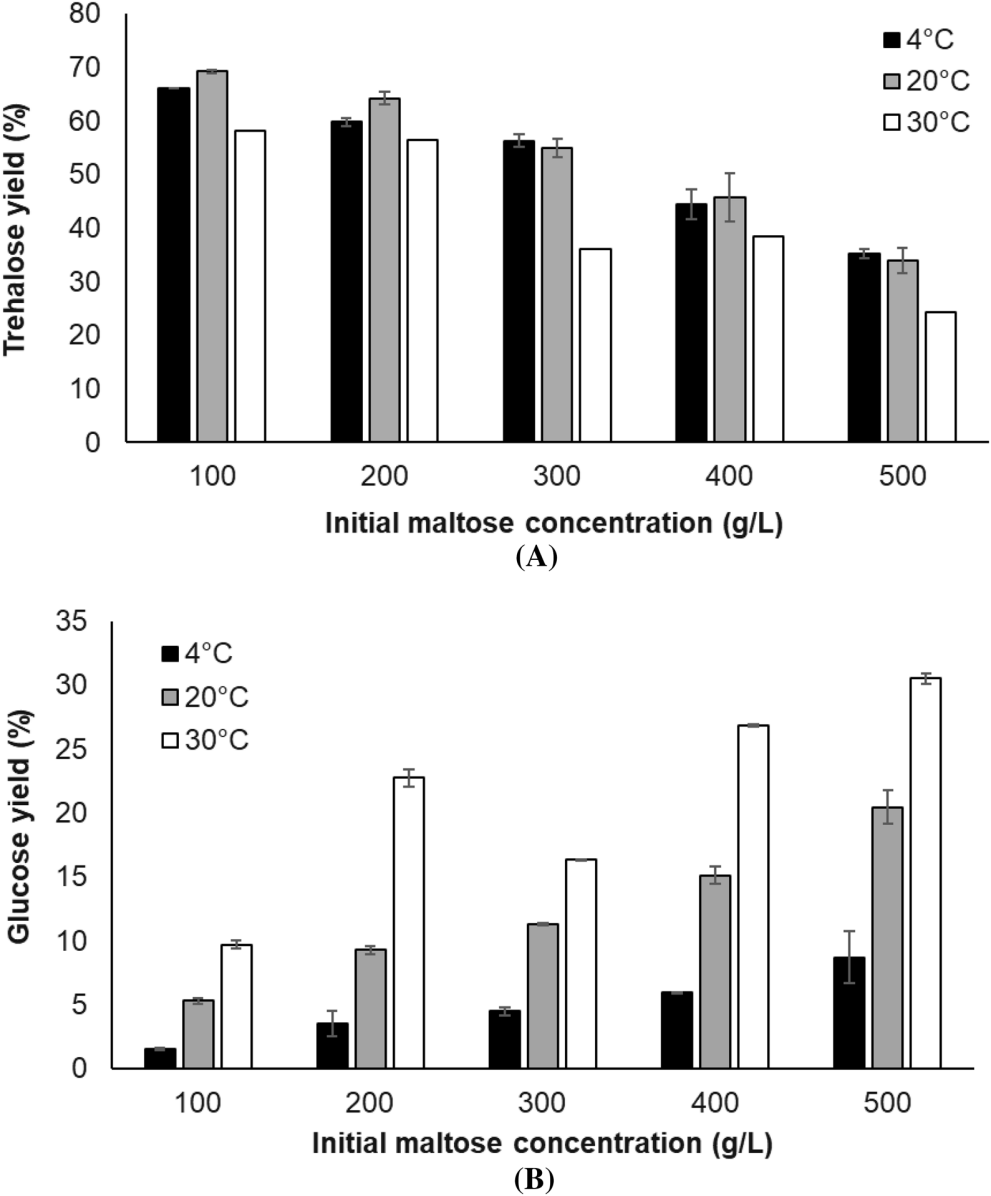
Effect of substrate concentration on the yield of trehalose by PaTreS. The reactions were carried out in 50 mM phosphate buffer (pH 7.0) at 4, 20, and 30 °C for 24 h. **A** Trehalose yield **B** Glucose yield

**Fig. 8:**
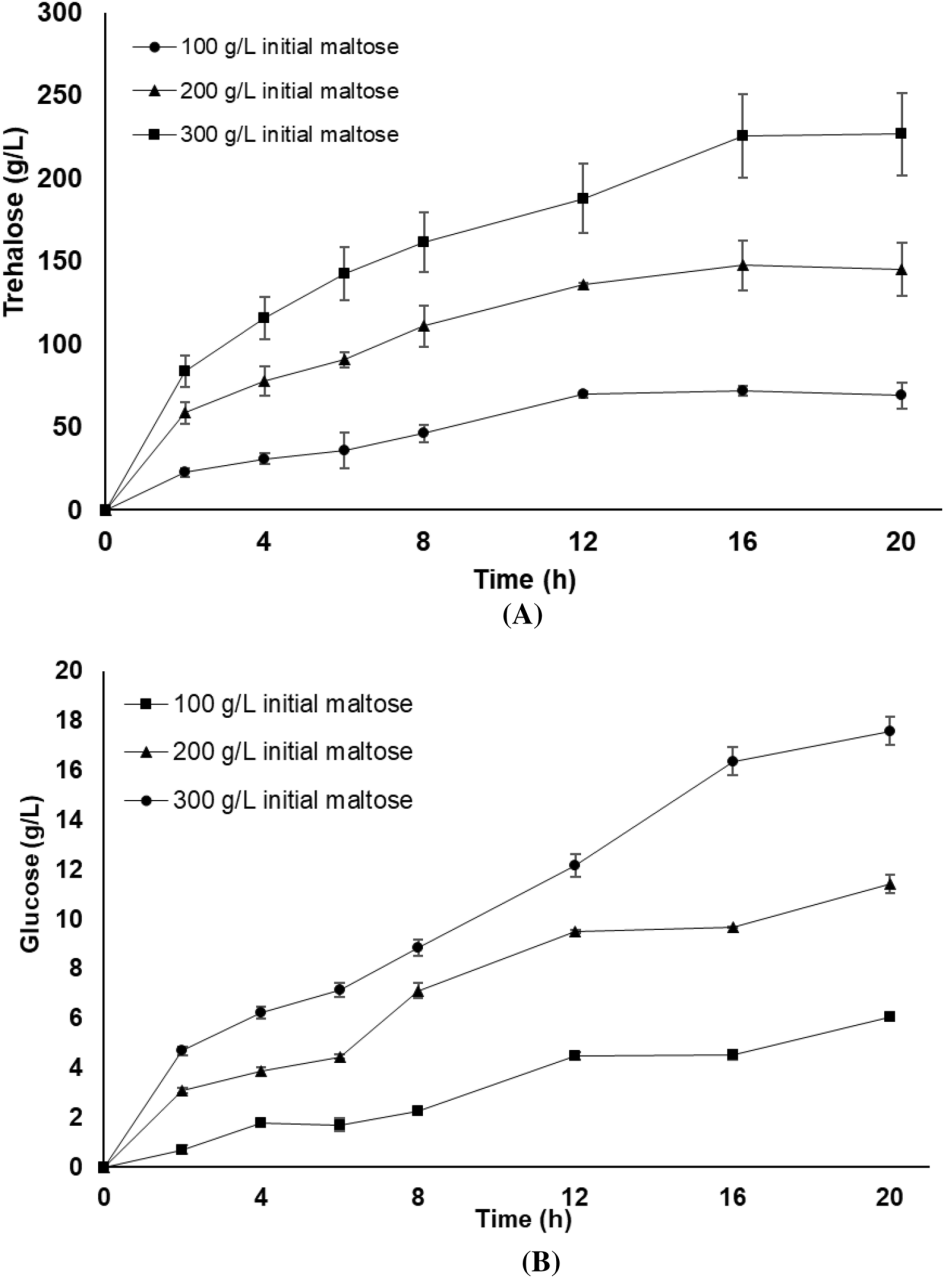
Production of trehalose from maltose by PaTreS. The conversion was carried out in phosphate buffer (50 mM, pH 7) at 20 °C for 20 h. **A** Trehalose yield **B** Glucose yield

The performance of PaTreS on trehalose biosynthesis was evaluated at varying substrate concentrations at 4, 20, and 30 °C (Fig. 7). The maximum conversion yield of 55.0–69.3% was achieved with 100–300 g/L maltose concentration at 4–20 °C. Maltose concentration higher than 300 g/L resulted in a lower trehalose yield with higher formation of glucose. A decrease in trehalose yield was also found at 30 °C, indicating that the enzyme has a strong specificity for trehalose at low temperatures. The time course of trehalose production was then studied using different maltose concentrations in a 50 mL reaction at pH 7, 20 °C for 20 h. The maximum trehalose concentrations of 71.7, 147.3, and 225.5 g/L, were achieved using 100, 200, and 300 g/L maltose as the substrate, respectively (Fig. 8). The concentration of glucose was between 4.5 and 16.4 g/L under these experimental conditions. This was equivalent to the trehalose yield between 55 and 69% with the maximal yield at 20 °C.

## Discussion

A recombinant TreS from *Pseudarthrobacter* sp. TBRC 2005 was first characterized in this study. The enzyme showed a high identity of 99% to an uncharacterized maltose alpha-D-glucosyltransferase from *Pseudarthrobacter* and *Arthrobacter* species. According to evolutionary analysis with the sequence data set of biochemically characterized TreS enzymes from Agarwal et al. (2021), PaTreS was classified into the same clade with TreS from glycoside hydrolase family 16 (GH13_16) (Additional file 1: Fig. S2 and Table S1). The enzyme shared a similar three-dimensional structure comprising 4 domains to other homologous enzymes with the presence of conserved key amino acid residues (H128/D225/E267/H334/D335) in the catalytic domain previously reported in other microbial TreS enzymes (Agarwal et al. 2021; Wang et al. 2017; Wang et al. 2014; Wang et al. 2007).

PaTreS was classified as a cold-active enzyme based on the definition of psychrophilic enzymes (Hamid et al. 2022; Joseph et al. 2008; Kuddus 2018; Maharana and Ray 2015; Santiago et al. 2016; Sarmiento et al. 2015). PaTreS showed high activity in broad temperature ranges (10–40 °C). The enzyme optimally worked at 20 °C with more than 80% and 60% residual at 10 °C and 4 °C, respectively under the experimental conditions. The working condition of PaTreS was similar to the optimum temperature (20 °C) of the characterized cold-active lipases from different microorganisms (Joseph et al. 2008). Comparative results with previous TreS enzymes that show optimum below 30 °C, PaTreS showed trehalose yield higher than those reported for TreS from *Thermobifida fusca* DSM 43792 (Wei et al. 2004) and *Rhodococcus opacus* ATCC 41021(Yan et al. 2013) but lower than TreS from *Deinococcus radiodurans* ATCC 13939 (Wang et al. 2007) and *Pimelobacter* sp R48 (Tsusaki et al 1996). PaTreS also showed higher thermostability better than its homolog in *D. radiodurans* ATCC 13939 (Wang et al. 2007) and *Pimelobacter* sp. R48 (Tsusaki et al. 1996). Although PaTreS showed strong activity at low temperature, it gave a higher trehalose yield than those reported by several mesophilic and thermophilic TreS enzyme such as *D. geothermalis* DSMZ 11300 (Filipkowski et al.^2012^), *Meiothermus ruber* (Zhu et al. 2010), *T. fusca* (DSM 43792) (Wei et al. 2004), *Corynebacterium glutamicum* ATCC 13032 (Kim et al. 2010), *D. radiodurans* R1 (Filipkowski et al. 2012), *Enterobacter hormaechei* (Yue et al. 2009), *Arthrobacter aurescens* CGMCC1.1892a (Wu et al. 2009), *Pseudomonas* sp. P8005 (Gao et al. 2013), which were in the range of 43.6–67.0%. Moreover, its byproduct (glucose) was lower than that obtained from thermophilic and mesophilic TreS from *D. geothermalis* DSMZ 11300 (Filipkowski et al. 2012), *T. fusca* (DSM 43792) (Wei et al. 2004), *Thermomonospora curvata* DSM 43183 (Liang et al. 2013), *C. glutamicum* (ATCC 13032) Kim et al. 2010, *D. radiodurans* R1 (Filipkowski et al. 2012), *Paenarthrobacter aurescens* CGMCC1.1892a (Wu et al. 2009), *Pseudomonas* sp. P8005 (Gao et al. 2013), which were between 7.3 and 21.7%.

Based on evolutionary analysis, PaTreS was classified into the same clade with TreS enzymes previously reported to be active at low temperatures, with the optimal temperature ranging from 20 to 37 °C (Additional file 1: Fig. S3). The temperature profile of PaTreS was similar to TreS from *Pimelobacter* sp. R48 (20 °C), *Thermobifida fusca* DSM 43792 (25 °C), *D. radiodurans* ATCC 13939 (15 °C) and *R. opacus* ATCC 41021 (25 °C) (Wu et al. 2009; Tsusaki et al. 1996; Wei et al. 2004; Yan et al. 2013). However, PaTreS showed higher relative activity at low temperature (≤ 20 °C) than the previous TreS enzymes from *Pimelobacter* sp. R48, *T. fusca* DSM 43792, *R. opacus* ATCC 41021 (Tsusaki et al. 1996; Wei et al. 2004; Yan et al. 2013). The low-temperature active characteristic of PaTreS could sresult from the lower percentage of nonpolar residues (Gly, Ala, Ile, Leu, Val, Phe, Pro, and Trp) (50.42%) than those found in thermophilic TreS from *Thermus thermophilus* (53.78%), *Thermus antranikianii* (53.91%), and *Meiothermus ruber* (53.01%) which exhibited the highest activity at 50–65 °C. The presence of non-polar residues in thermophilic proteins could enhance the protein structural stability by creating hydrophobic interactions which can stabilize the protein folding configuration (Panja et al. 2015). Moreover, the lower distribution of Asp, Arg, Glu, and Lys residues and salt bridge formation in PaTreS could be another factor contributing to its activity at low temperatures (Agarwal et al. 2021; Panja et al. 2015).

In general, TreS can reversibly interconvert maltose and trehalose in a reaction (Cai et al. 2018). The *V*_*max*_ and *K*_*m*_ of PaTreS showed a stronger affinity for maltose and a preferred reaction direction for trehalose synthesis. Moreover, the catalytic efficiency (k_*cat*_/K_*m*_) of PaTreS is higher than the previously reported cold-active TreS enzymes from *R. opacus* (Yan et al. 2013). The higher preference and catalytic reaction rate of the enzyme against maltose led to a higher conversion rate from maltose to trehalose.

The recombinant PaTreS showed the optimum pH at pH 7.0 but it had high stability in the pH range from 7.0 to 9.0. The wide pH range of PaTreS was similar to the previous TreS from *C. glutamicum*, *M. ruber*, *P. torridus*, *P. putida*, *R. opacus*, and *T. aquaticus* (Kim et al. 2010; Zhu et al. 2010; Chen et al. 2006; Ma et al. 2006; Yan et al. 2013; Nishimoto et al. 1996). In addition, PaTreS showed higher stability at alkaline pH than those of the previously reported TreS, which showed 66% residual activity at pH 10. Higher stability under alkaline condition of PaTreS may be due to the ionization state of amino acid residues in the protein structure which remain unchanged at high pH. This leads to the maintained protein shape and enzyme activity under alkaline conditions (Di et al. 2012).

Application of PaTreS on trehalose biosynthesis using high concentration maltose syrup (100–300 g/L) led to high trehalose yield (71.7–225.5 g/L), equivalent to 55–69% conversion with low formation of glucose byproduct (4.5–16.4 g/L). In comparison with previously reported trehalose synthases, PaTreS has ability to convert higher maltose concentration compared to that reported for TreS from *Corynebacterium glutamicum* ATCC 13032 (5 g/L maltose, 3.45 g/L trehalose) (Kim et al. 2010), hot spring metagenome (36.03 g/L maltose, 27 g/L trehalose) (Agarwal et al. 2021), *Pseudomonas putida* KT2440 (150 g/L maltose, 92.2 g/L trehalose) (Zheng et al. 2015), and *Pseudomonas monteilii* TBRC 1196 (200 g/L maltose, 118.2 g/L trehalose) (Trakarnpaiboon et al. 2021). However, the obtained trehalose yield was lower than that reported from a TreS from saline–alkali soil metagenomes gave 234 g/L trehalose from 300 g/L maltose under mesophilic alkaline conditions (45 °C, pH 9.0) (Jiang et al. 2013). However, considering operation cost, the use of PaTreS could be advantageous in terms of lower heating energy cost and reduced risk of contamination (Hamid et al. 2022; Kuddus 2018). Therefore, as a result, the recombinant PaTreS provides a potential candidate for large-scale bioconversion of trehalose from high substrate loading at low temperatures (4–20 °C) and a wide pH range (6.0–8.0).

## Conclusion

This study described the cold-active trehalose synthase (PaTreS) from *Pseudarthrobacter* sp. TBRC 2005. Due to its low optimum temperature with high substrate and product specificity, and kinetic characteristics, PaTreS is one of the most promising biocatalysts for converting high concentration maltose syrup to trehalose. The enzymatic conversion process demonstrated a high conversion yield and product specificity under low-temperature settings which are beneficial for low byproduct generation and reduced risk of microbial contamination.

### Supplementary Information

**Additional file 1: Figure S1.** Evolutionary analysis of PaTreS and homolog sequences classified in GH13 subfamily GH13_16 and GH13_33 subfamilies. **Figure S2.** Fiver conserved regions found in PaTreS compared with trehalose synthases from GH13_16 and GH13_33 subfamilies. **Figure S3.** Evolutionary analysis of PaTreS and biochemically characterized trehalose synthases. **Table S1.** List of trehalose synthases from GH13_16 and GH13_33 subfamilies used in conserved region analysis

## Data Availability

All data generated or analyzed during this study are included in this published article (and its supplementary information files).
