# Supplementary material for: Characterization of cold-active trehalose synthase from Pseudarthrobacter sp. for trehalose bioproduction
Source: Bioresour Bioprocess. 2023 Sep 25;10(1):65. doi: 10.1186/s40643-023-00681-0 (PMC10992939; doi:10.1186/s40643-023-00681-0)
Supplement: Supplementary file 1 — Additional file 1: Figure S1. Evolutionary analysis of PaTreS and homolog sequences classified in GH13 subfamily GH13_16 and GH13_33 subfamilies. Figure S2. Fiver conserved regions found in PaTreS compared with trehalose synthases from GH13_16 and GH13_33 subfamilies. Figure S3. Evolutionary analysis of PaTreS and biochemically characterized trehalose synthases. Table S1. List of trehalose synthases from GH13_16 and GH13_33 subfamilies used in conserved region analysis [file 40643_2023_681_MOESM1_ESM.docx]

**Additional Material**

**Characterization of cold-active trehalose synthase from *Pseudarthrobacter* sp. for trehalose bioproduction**

Srisakul Trakarnpaiboon^1^, Benjarat Bunterngsook^1^, Hataikarn Lekakarn^2^, Daran Prongjit^2^, Verawat Champreda^1*^

^1^Enzyme Technology Research Team, Biorefinery Technology and Bioproduct Research Group, National Center for Genetic Engineering and Biotechnology, 113 Thailand Science Park, Phahonyothin Road, Khlong Nueang, Khlong Luang, Pathumthani 12120, Thailand

^2^Department of Biotechnology, Faculty of Science and Technology, Thammasat University, Rangsit Campus, Khlong Nueang, Khlong Luang, Pathumthani 12120, Thailand

* Corresponding author. Address: National Center for Genetic Engineering and Biotechnology, 113 Thailand Science Park, Phahonyothin Road, Khlong Luang, Pathumthani 12120, Thailand. Tel.: +66 2564 6700 x 3446; fax: +66 2564 6707.

E-mail address: verawat@biotec.or.th (Verawat Champreda).

ORCID ID: 0000-0001-7768-1340

**
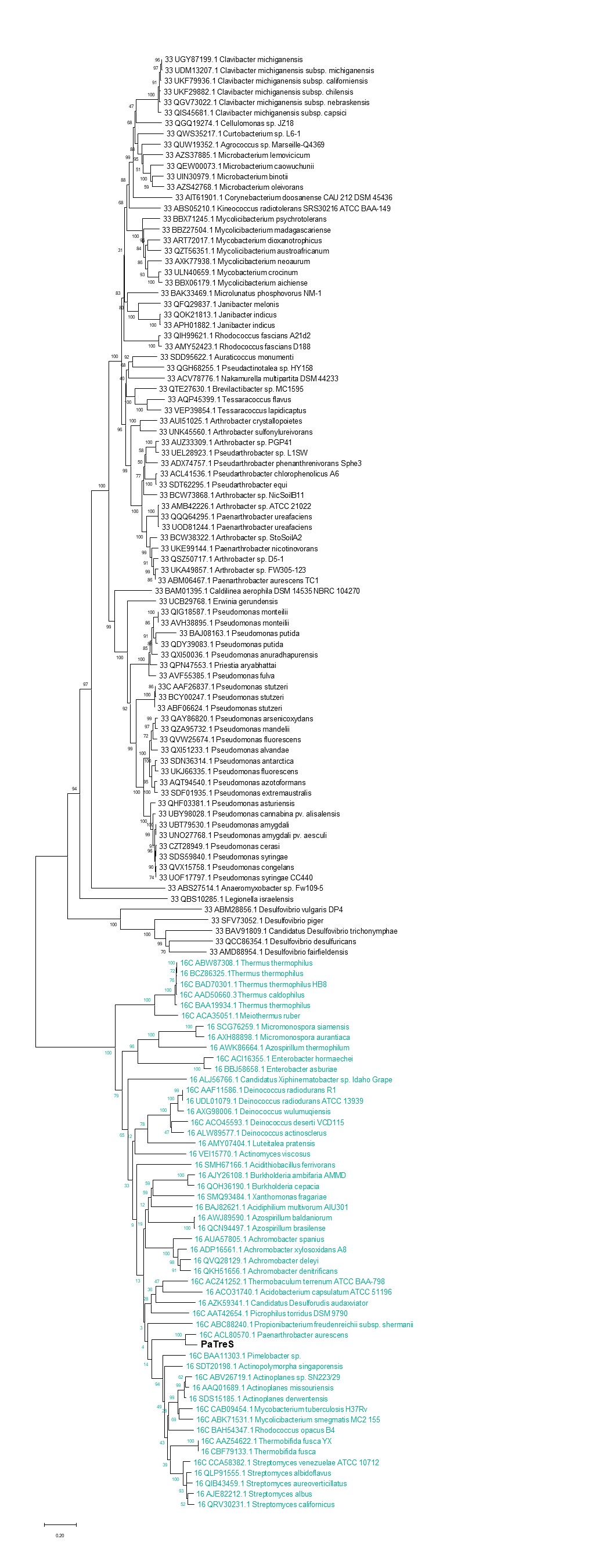
**

**Fig. S1** Evolutionary analysis of PaTreS and homolog sequences classified in GH13 subfamily GH13_16 and GH13_33 subfamilies (Table S1).


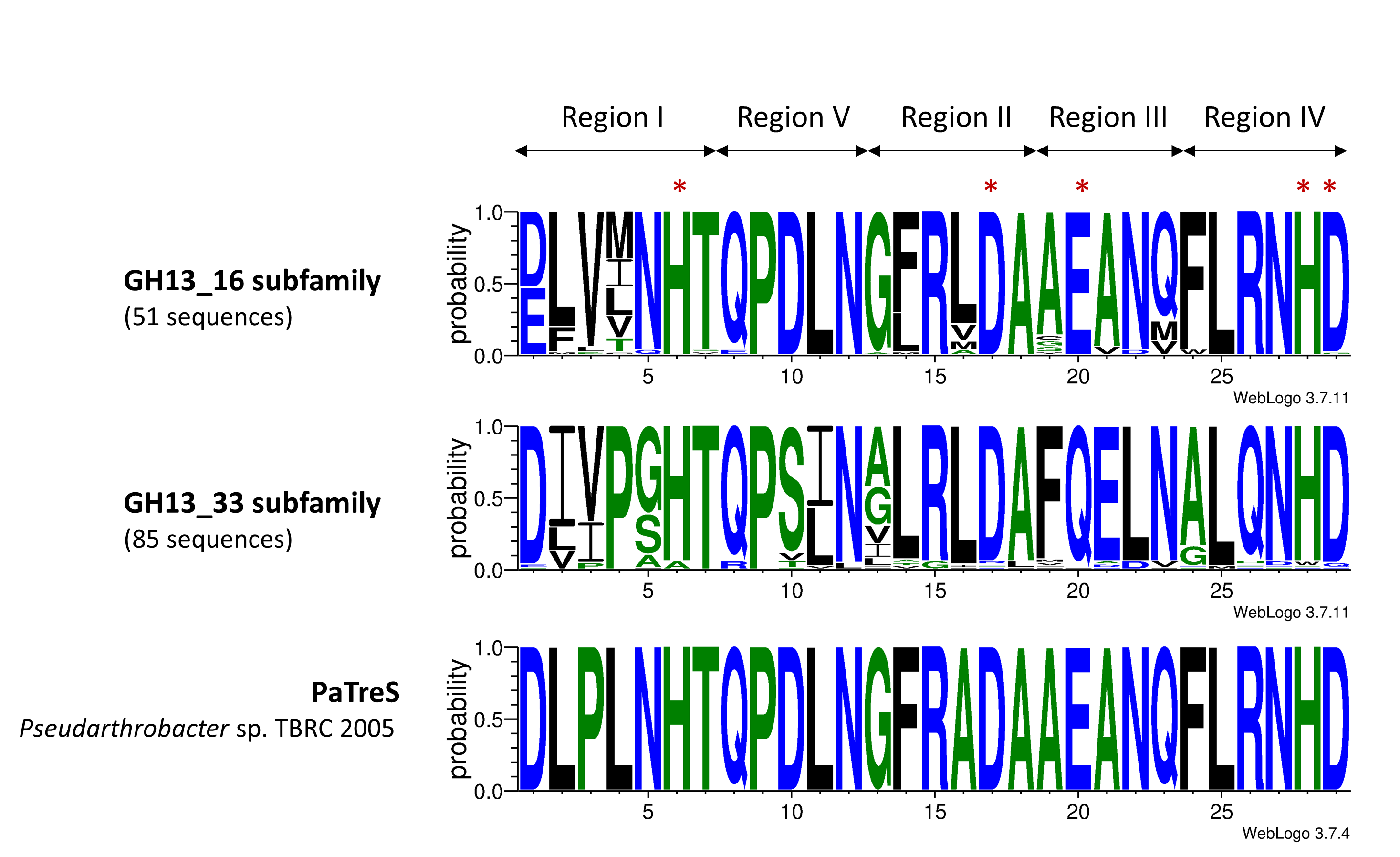


**Fig. S2** Fiver conserved regions found in PaTreS compared with trehalose synthases from GH13_16 and GH13_33 subfamilies (Table S1). The key amino acids are marked with the asterisks.


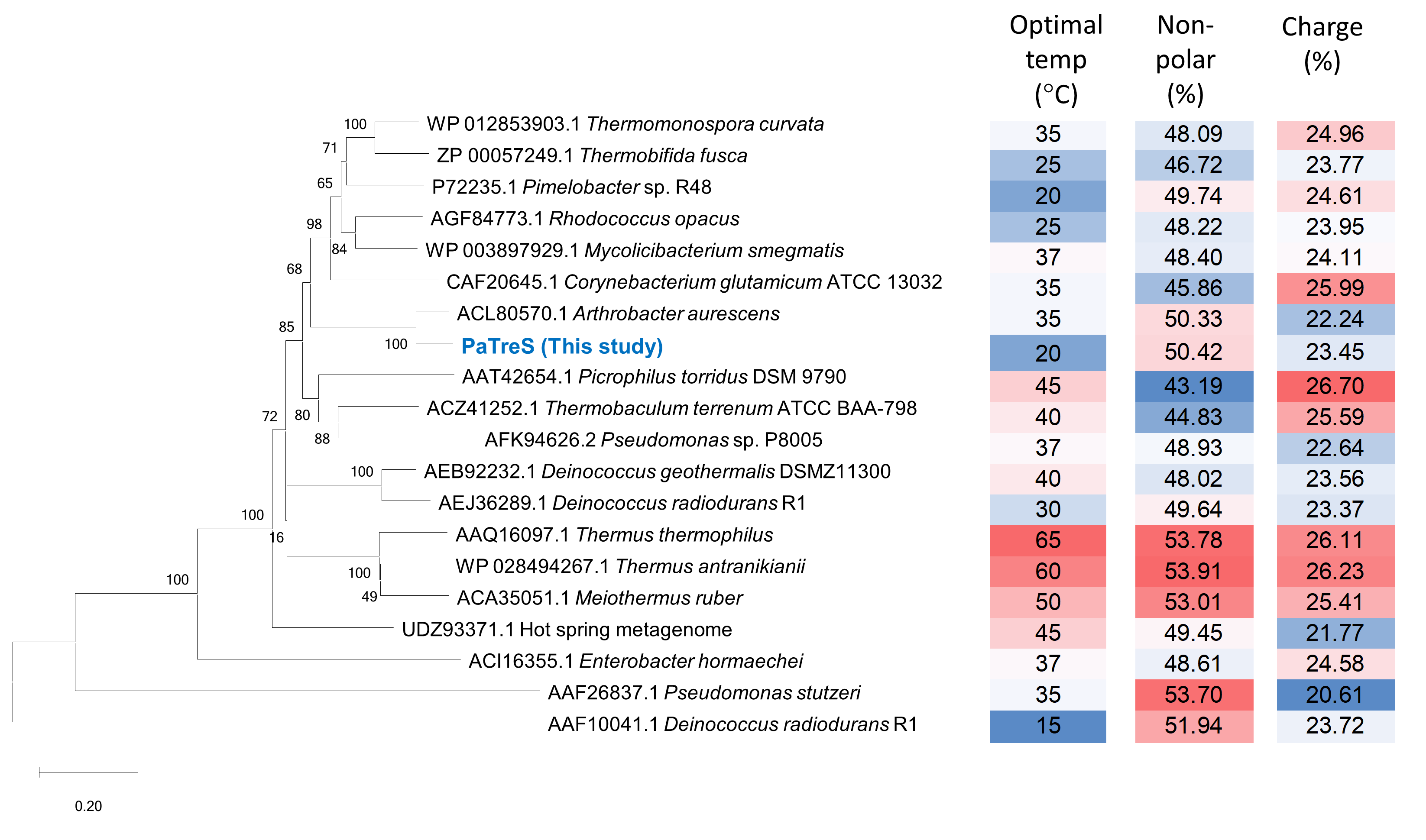


**Fig. S3** Evolutionary analysis of PaTreS and biochemically characterized trehalose synthases. The optimal temperature and percentage of non-polar and charge residues were presented as heatmap color diagram.

**Table S1.** List of trehalose synthases from GH13_16 and GH13_33 subfamilies used in conserved region analysis

| **Subfamily** | **Accession no.** | **Species** |
| --- | --- | --- |
| 16 | AAT42654.1 | *Picrophilus torridus* DSM 9790 |
| 16 | ABV26719.1 | *Actinoplanes sp.* |
| 16 | ACO45593.1 | *Deinococcus deserti* |
| 16 | AAF11586.1 | *Deinococcus radiodurans* |
| 16 | ACI16355.1 | *Enterobacter hormaechei* |
| 16 | ACA35051.1 | *Meiothermus ruber* |
| 16 | CAB09454.1 | *Mycobacterium tuberculosis* |
| 16 | ABK71531.1 | *Mycolicibacterium smegmatis* |
| 16 | ACL80570.1 | *Paenarthrobacter aurescens* |
| 16 | BAA11303.1 | *Pimelobacter sp.* |
| 16 | ABC88240.1 | *Propionibacterium freudenreichii subsp. Shermanii* |
| 16 | BAH54347.1 | *Rhodococcus opacus* |
| 16 | CCA58382.1 | *Streptomyces venezuelae* ATCC 10712 |
| 16 | ACZ41252.1 | *Thermobaculum terrenum* ATCC BAA-798 |
| 16 | AAZ54622.1 | *Thermobifida fusca* |
| 16 | BAA19934.1 | *Thermus thermophilus* |
| 16 | AAD50660.3 | *Thermus caldophilus* |
| 16 | BAD70301.1 | *Thermusthermophilus* |
| 16 | ABW87308.1 | *Thermus thermophilus* |
| 16 | QVQ28129.1 | *Achromobacter deleyi* |
| 16 | QKH51656.1 | *Achromobacter denitrificans* |
| 16 | AUA57805.1 | *Achromobacter spanius* |
| 16 | ADP16561.1 | *Achromobacter xylosoxidans* |
| 16 | BAJ82621.1 | *Acidiphilium multivorum* |
| 16 | SMH67166.1 | *Acidithiobacillus ferrivorans* |
| 16 | AMY07404.1 | *Luteitalea pratensis* |
| 16 | ACO31740.1 | *Acidobacterium capsulatum* |
| 16 | VEI15770.1 | *Actinomyces viscosus* |
| 16 | SDS15185.1 | *Actinoplanes derwentensis* |
| 16 | AAQ01689.1 | *Actinoplanes missouriensis* |
| 16 | SDT20198.1 | *Actinopolymorpha singaporensis* |
| 16 | AWJ89590.1 | *Azospirillum baldaniorum* |
| 16 | QCN94497.1 | *Azospirillum brasilense* |
| 16 | AWK86664.1 | *Azospirillum thermophilum* |
| 16 | AJY26108.1 | *Burkholderia ambifaria* |
| 16 | QOH36190.1 | *Burkholderia cepacia* |
| 16 | AZK59341.1 | *Candidatus Desulforudis audaxviator* |
| 16 | ALJ56766.1 | *Candidatus Xiphinematobacter sp.* |
| 16 | ALW89577.1 | *Deinococcus actinosclerus* |
| 16 | UDL01079.1 | *Deinococcus radiodurans* ATCC 13939 |
| 16 | AXG98006.1 | *Deinococcus wulumuqiensis* |
| 16 | BBJ58658.1 | *Enterobacter asburiae* |
| 16 | SCG76259.1 | *Micromonospora siamensis* |
| 16 | AXH88898.1 | *Micromonospora aurantiaca* |
| 16 | QLP91555.1 | *Streptomyces albidoflavus* |
| 16 | AJE82212.1 | *Streptomyces albus* |
| 16 | QIB43459.1 | *Streptomyces aureoverticillatus* |
| 16 | QRV30231.1 | *Streptomyces californicus* |
| 16 | CBF79133.1 | *Thermobifida fusca* |
| 16 | BCZ86325.1 | *Thermus thermophilus* |
| 16 | SMQ93484.1 | *Xanthomonas fragariae* |
| 33 | AAF26837.1 | *Pseudomonas stutzeri* |
| 33 | QUW19352.1 | *Agrococcus sp.* |
| 33 | ABS27514.1 | *Anaeromyxobacter sp.* |
| 33 | AUI51025.1 | *Arthrobacter crystallopoietes* |
| 33 | AMB42226.1 | *Arthrobacter sp.* ATCC 21022 |
| 33 | QSZ50717.1 | *Arthrobacter sp.* |
| 33 | UKA49857.1 | *Arthrobacter sp.* |
| 33 | BCW73868.1 | *Arthrobacter sp.* |
| 33 | AUZ33309.1 | *Arthrobacter sp.* |
| 33 | BCW38322.1 | *Arthrobacter sp.* |
| 33 | UNK45560.1 | *Arthrobacter sulfonylureivorans* |
| 33 | SDD95622.1 | *Auraticoccus monumenti* |
| 33 | QTE27630.1 | *Brevilactibacter sp.* |
| 33 | BAM01395.1 | *Caldilinea aerophila* DSM 14535 |
| 33 | BAV91809.1 | *Candidatus Desulfovibrio trichonymphae* |
| 33 | QGQ19274.1 | *Cellulomonas sp.* |
| 33 | UGY87199.1 | *Clavibacter michiganensis* |
| 33 | UKF79936.1 | *Clavibacter michiganensis subsp. Californiensis* |
| 33 | QIS45681.1 | *Clavibacter michiganensis subsp. Capsici* |
| 33 | UKF29882.1 | *Clavibacter michiganensis subsp. Chilensis* |
| 33 | UDM13207.1 | *Clavibacter michiganensis subsp. Michiganensis* |
| 33 | QGV73022.1 | *Clavibacter michiganensis subsp. Nebraskensis* |
| 33 | AIT61901.1 | *Corynebacterium doosanense* |
| 33 | QWS35217.1 | *Curtobacterium sp.* |
| 33 | QCC86354.1 | *Desulfovibrio desulfuricans* |
| 33 | AMD88954.1 | *Desulfovibrio fairfieldensis* |
| 33 | SFV73052.1 | *Desulfovibrio piger* |
| 33 | ABM28856.1 | *Desulfovibrio* |
| 33 | UCB29768.1 | *Erwinia gerundensis* |
| 33 | QOK21813.1 | *Janibacter indicus* |
| 33 | QFQ29837.1 | *Janibacter melonis* |
| 33 | APH01882.1 | *Janibacter indicus* |
| 33 | ABS05210.1 | *Kineococcus radiotolerans* |
| 33 | QBS10285.1 | *Legionella israelensis* |
| 33 | UIN30979.1 | *Microbacterium binotii* |
| 33 | QEW00073.1 | *Microbacterium caowuchunii* |
| 33 | AZS37885.1 | *Microbacterium lemovicicum* |
| 33 | AZS42768.1 | *Microbacterium oleivorans* |
| 33 | BAK33469.1 | *Microlunatus phosphovorus* |
| 33 | ULN40659.1 | *Mycobacterium crocinum* |
| 33 | ART72017.1 | *Mycobacterium dioxanotrophicus* |
| 33 | BBX06179.1 | *Mycolicibacterium aichiense* |
| 33 | QZT56351.1 | *Mycolicibacterium austroafricanum* |
| 33 | BBZ27504.1 | *Mycolicibacterium madagascariense* |
| 33 | AXK77938.1 | *Mycolicibacterium neoaurum* |
| 33 | BBX71245.1 | *Mycolicibacterium psychrotolerans* |
| 33 | ACV78776.1 | *Nakamurella multipartita* DSM 44233 |
| 33 | ABM06467.1 | *Paenarthrobacter aurescens* |
| 33 | UKE99144.1 | *Paenarthrobacter nicotinovorans* |
| 33 | UOD81244.1 | *Paenarthrobacter ureafaciens* |
| 33 | QQQ64295.1 | *Paenarthrobacter ureafaciens* |
| 33 | QPN47553.1 | *Priestia aryabhattai* |
| 33 | QGH68255.1 | *Pseudactinotalea sp.* |
| 33 | ACL41536.1 | *Pseudarthrobacter chlorophenolicus* |
| 33 | SDT62295.1 | *Pseudarthrobacter equi* |
| 33 | ADX74757.1 | *Pseudarthrobacter phenanthrenivorans* |
| 33 | UEL28923.1 | *Pseudarthrobacter sp.* |
| 33 | QXI51233.1 | *Pseudomonas alvandae* |
| 33 | UBT79530.1 | *Pseudomonas amygdali* |
| 33 | UNO27768.1 | *Pseudomonas amygdali* |
| 33 | SDN36314.1 | *Pseudomonas antarctica* |
| 33 | QXI50036.1 | *Pseudomonas anuradhapurensis* |
| 33 | QAY86820.1 | *Pseudomonas arsenicoxydans* |
| 33 | QHF03381.1 | *Pseudomonas asturiensis* |
| 33 | AQT94540.1 | *Pseudomonas azotoformans* |
| 33 | UBY98028.1 | *Pseudomonas cannabina* |
| 33 | CZT28949.1 | *Pseudomonas cerasi* |
| 33 | QVX15758.1 | *Pseudomonas congelans* |
| 33 | SDF01935.1 | *Pseudomonas extremaustralis* |
| 33 | UKJ66335.1 | *Pseudomonas fluorescens* |
| 33 | QVW25674.1 | *Pseudomonas fluorescens* |
| 33 | AVF55385.1 | *Pseudomonas fulva* |
| 33 | QZA95732.1 | *Pseudomonas mandelii* |
| 33 | QIG18587.1 | *Pseudomonas monteilii* |
| 33 | AVH38895.1 | *Pseudomonas monteilii* |
| 33 | BAJ08163.1 | *Pseudomonas putida* |
| 33 | QDY39083.1 | *Pseudomonas putida* |
| 33 | ABF06624.1 | *Pseudomonas stutzeri* |
| 33 | BCY00247.1 | *Pseudomonas stutzeri* |
| 33 | SDS59840.1 | *Pseudomonas syringae* |
| 33 | UOF17797.1 | *Pseudomonas syringae* |
| 33 | QIH99621.1 | *Rhodococcus fascians* |
| 33 | AMY52423.1 | *Rhodococcus fascians* |
| 33 | AQP45399.1 | *Tessaracoccus flavus* |
| 33 | VEP39854.1 | *Tessaracoccus lapidicaptus* |
